# Actinic cheilitis and lip squamous cell carcinoma: Literature review and new data from Brazil

**DOI:** 10.4317/jced.55133

**Published:** 2019-01-01

**Authors:** Fernanda-Weber Mello, Gilberto Melo, Filipe Modolo, Elena-Riet-Correa Rivero

**Affiliations:** 1 Postgraduate Program in Dentistry, Federal University of Santa Catarina, Florianópolis, Santa Catarina, Brazil; 2 Department of Pathology, Federal University of Santa Catarina, Florianópolis, Santa Catarina, Brazil

## Abstract

**Background:**

To investigate the prevalence of malignant and potentially malignant lesions of the lip in an oral pathology service and to compare these data with a literature review.

**Material and Methods:**

A total of 3173 biopsy reports and histopathological records were analyzed. Cases with a histological diagnosis of actinic cheilitis (AC) with or without epithelial dysplasia, in situ carcinoma, or lip squamous cell carcinoma (LSCC) were included. A comprehensive literature review was conducted to investigate the prevalence of AC and/or LSCC.

**Results:**

124 cases (3.91%) were included, 75 (60.5%) had some degree of epithelial dysplasia and 31 (25.0%) were LSCC. Clinically, most of the lesions were diagnosed as AC (50.8%); however, eight cases clinically reported as AC were histologically diagnosed as LSCC. Regarding clinical characteristics, most individuals were fair-skinned male, with mean age of 54.3±12.3 years, and with a history of long-term solar exposure. Furthermore, 18 articles were selected from the literature, showing that the lower lip was predominantly affected and that most individuals were males, fair-skinned, and older than 40 years.

**Conclusions:**

Since most of the cases diagnosed clinically as AC presented some degree of epithelial dysplasia, it is important to emphasize the value of biopsy and the histological evaluation of this lesion.

** Key words:**Actinic cheilitis, Precancerous conditions, In situ carcinoma, Oral diagnosis, Mouth neoplasms.

## Introduction

Oral cancer is one of the most frequent malignant neoplasia among Brazilian individuals, this malignancy is the fifth more frequent among Brazilian males and the twelfth among Brazilian females (1). In 2018, 11.2 new cases were estimated per 100.000 male Brazilians (1); furthermore, the survival rate of this disease is approximately 50% in five years (2). Considering only lip squamous cell carcinoma (LSCC), the survival rate increases; however, the treatment can have several consequences, especially whenever surgery is necessary, since it can cause deformation and, consequently, affect the patient’s quality of life (3). Frequently, lip potentially malignant lesions (LPML) precede LSCC. The most important LPML is actinic cheilitis (AC), characterized by variable degrees of alterations in the epithelial lining of the lips, caused mainly by long-term and chronic exposition to sunlight (4). Clinically, AC may present lip atrophy, dryness, erythema, and indistinct vermillion border (5).

The main etiological factor associated with these lesions is the long term exposure to ultraviolet (UV) rays without any protection (6). Therefore, outdoor workers, such as fisherman and farmers, submitted to long periods of solar exposure are vulnerable to developing LPML and LSCC. Since melanin is a natural protection against UV rays, fair-skinned people are the most affected by these disorders (7). Furthermore, the possible carcinogenic effect of factors associated with lifestyle behaviors, such as tobacco and alcohol consumption on lip carcinogenesis remains unclear. However, some authors suggested that the development of LPML and LSCC relies mostly on UV exposure than on secondary behavior habits (8).

In the state of Santa Catarina/Brazil, approximately 15% of the population lives in rural areas and 84% of the population above 15 years-old is composed of fair-skinned individuals (9). In addition, some areas of this state are highly affected by UV radiation, with an average of 6 in the UV index of the World Health Organization (WHO) (10). In the summer season, this rate can increase, resulting in more sun damage to the lip and skin areas (11). Consequently, Santa Catarina has one of the highest rates of skin cancer in Brazil (1). Considering that the main etiological factor for skin cancer is prolonged UV exposure, this population may be at risk of developing LSCC and AC as well. Therefore, the aim of this descriptive study was to analyze the prevalence of LSCC and AC in an oral pathology service in Brazil, to describe the individuals’ characteristics and associated risk factors reported, as well as their histological diagnosis. Moreover, the data were also compared with those obtained from a review of the literature.

## Material and Methods

-Main study (MS)

Biopsy reports and histopathological records from March 2007 to February 2018 were analyzed, totalizing 3173 cases. This study was approved by the Ethics Committee of the authors’ institution of affiliation (No 1.097.375; CAAE: 42095715.1.0000.0121). Firstly, biopsy reports were analyzed and lip lesions were selected (only lesions in the lip vermillion semi-mucosa were considered). Then, cases that were clinically diagnosed as AC, leukoplakia, erythroplakia, erythroleukoplakia, in situ carcinoma, and LSCC were selected. Lastly, histopathological records were evaluated and cases were included in the sample when histologically diagnosed as AC with no epithelial dysplasia (NoED), AC with mild epithelial dysplasia (MiED), AC with moderated epithelial dysplasia (MoED), AC with severe epithelial dysplasia (SED), in situ carcinoma (ISC), and lip squamous cell carcinoma and LSCC. Cases that did not fulfill these criteria were excluded from the sample.

From the biopsy reports, information of clinical diagnosis, gender, age and skin color of the patients, lesion location, smoking and/or alcohol consumption habits, and history of chronic solar exposure was collected. Individuals that had outdoor occupations or self-reported periodical outdoor activities with long duration were considered as with “prolonged solar exposure”. All clinical data of patients were collected from biopsy files and, therefore, were reported by the responsible surgeon of each procedure. Furthermore, histological diagnosis was performed following criteria of the WHO (12) by an oral pathology team, composed by three experienced oral pathologists. After selecting the sample, data were tabulated on Excel 2016 (Microsoft Office 2016, Microsoft) and analyzed using the statistical software SPSS Statistics 21 (IBM Corp., Armonk, NY, USA). Mann-Whitney test was performed to compare age between AC and LSCC groups. A two-way Chi-square test and Fisher exact test were applied to compare variables of interest in AC and LSCC groups. The statistical significance was fixed at α=0.05.

-Literature review (LR)

A comprehensive review of the literature was conducted to assess studies that investigated the prevalence of AC and/or LSCC. No time restriction was applied. Retrospective studies and case-series were considered eligible for inclusion. The following exclusion criteria were applied: 1) studies that did not report the histological diagnosis; 2) studies not published in English; and 3) full-text not available.

Electronic search strategies were developed and adapted for each of the following bibliographic databases: Embase, PubMed, and Web of Science. In addition, a grey literature search was performed in Google Scholar, ProQuest Dissertations & Thesis, and OpenGrey. The following truncations and word combinations summarize the search strategy: (prevalence OR prevalences OR occurrence OR frequency OR frequencies OR incidence OR epidemiology OR epidemiologic OR incidence OR incidences) AND (“actinic cheilitis” OR “actinic cheilosis” OR “actinic elastosis” OR “solar cheilitis” OR “solar cheilosis” OR “solar elastosis” OR “in situ carcinoma” OR “squamous cell carcinoma” OR “epidermoid carcinoma” OR “planocellular carcinoma” OR “squamous carcinoma”) AND (lip OR lips OR labium OR labial). All searches were conducted from the starting coverage date through 26 February 2018. A reference manager (EndNote X7, Thomson Reuters) was used to collect references and remove duplicates.

A two-phase process was performed in the selection of studies. In phase-1, titles and abstracts of identified references were screened for potentially eligible studies. In phase-2, a full-text read was performed and studies that met eligibility criteria were included. The following data were extracted from the articles included in the LR: author, year of publication, country, sample size, participants’ gender and age, histological diagnosis, and associated factors.

## Results

-Main study (MS)

**Figure 1:**
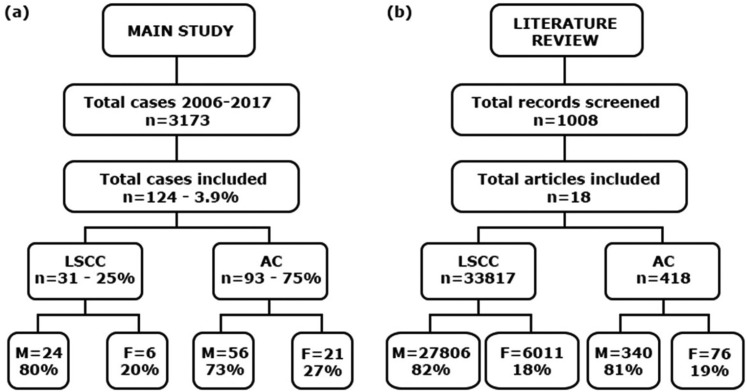
Flow chart of included cases (a) and literature review (b). Legend: AC, actinic cheilitis; F, female; LSCC, lip squamous cell carcinoma; M, male; n, number.

A total of 3173 cases of the pathology laboratory cases were analyzed; 124 files from 107 patients were selected to comprise the sample (Fig. 1a). Moreover, 16 individuals had more than one file in the laboratory archive, of which 8 had lesions with the same histological diagnosis in the second biopsy and 4 had higher degrees of epithelial dysplasia in the second sample; only one case evolved from AC to LSCC. Most patient were male (73.2%), fair-skinned (83.2%), and the mean age was 54.3±12.3 years. 103 files provided data regarding profession of individuals, from which the most frequent were farmers (18.5%), construction workers (11.6%), fishermen (9.7%), and drivers (7.8%).

There were few variations in the epidemiological profile of AC and LSCC patients. LSCC individuals tend to be older than AC individuals since the mean age of LSCC individuals was 56.3±12.2 years (ranging from 32-79 years) and in the AC group was 53.7±12.1 years (ranging from 24-79 years). The sex and skin color distribution were similar in both groups, and the concentration of individuals with chronical solar exposure was similar in AC than in LSCC group. 120 (96.8%) lesions were located in the lower lip and 04 (3.2%) in the upper lip. The lesions located in the upper lip were histologically diagnosed as NoED (n=1), AC with MiED (n=2), and LSCC (n=1). There was no statistic difference in regards to age, gender, skin color, solar exposure, and alcohol consumption between LSCC and AC groups (*p*>0.05). There was an association between smoking habits and histological diagnosis, the LSCC group presented higher rates of smoking habits than the AC group (*p*=0.03). The majority (80.64%) of AC cases presented some degree of epithelial dysplasia and the majority of LSCC cases were well and moderately differentiated in the histopathological analysis ( Table 1).

**Table 1:**
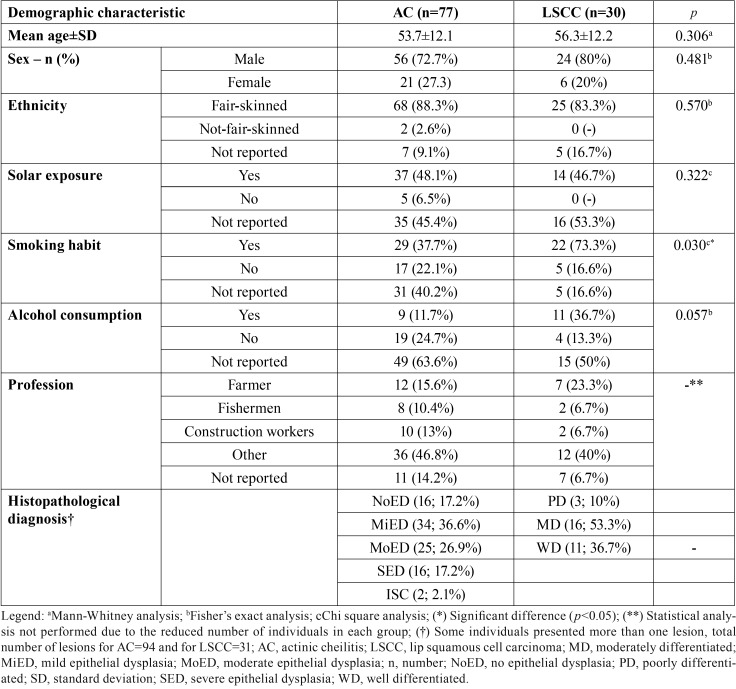
Characteristics of AC and LSCC cases.

Clinically, 62 (50%) lesions were diagnosed as AC, 19 (15.3%) as leukoplakia, and 20 (16.1%) as LSCC. Considering the histological features of these cases, 31 (25%) were SCC, two (1.6%) consisted of ISC, 77 (62.1%) were AC with some degree of epithelial dysplasia, and 16 (12.9%) were described as AC without epithelial dysplasia. 18 lesions clinically diagnosed as LSCC were squamous cell carcinomas at histological analysis, one was MiED, and one was MoED. In addition, 8 cases were initially described as AC by the surgeon and later diagnosed as LSCC by the oral pathology team ( Table 2).

**Table 2:**
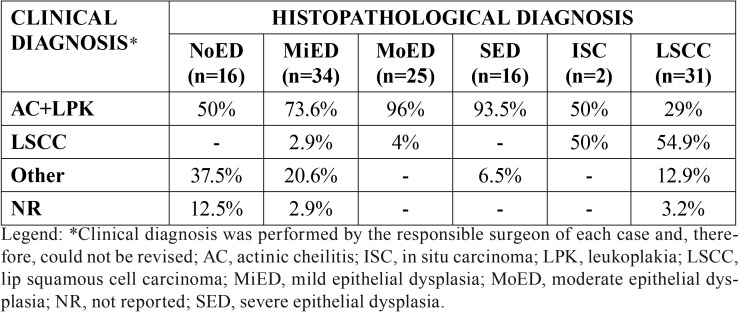
Correlation between clinical and histopathological diagnosis.

-Literature review (LR)

From the main electronic databases searches, 1590 papers were identified, of which 1008 remained after duplicates had been removed. After title and abstract screening, 49 articles were considered eligible for full-text reading, of which 16 met the inclusion criteria and were finally included. Moreover, 2 additional papers were included from the grey literature, resulting in 18 included studies (13-31) (Fig. 1b). The detailed information regarding data collected from each included study is provided in  Table 3.

**Table 3:**
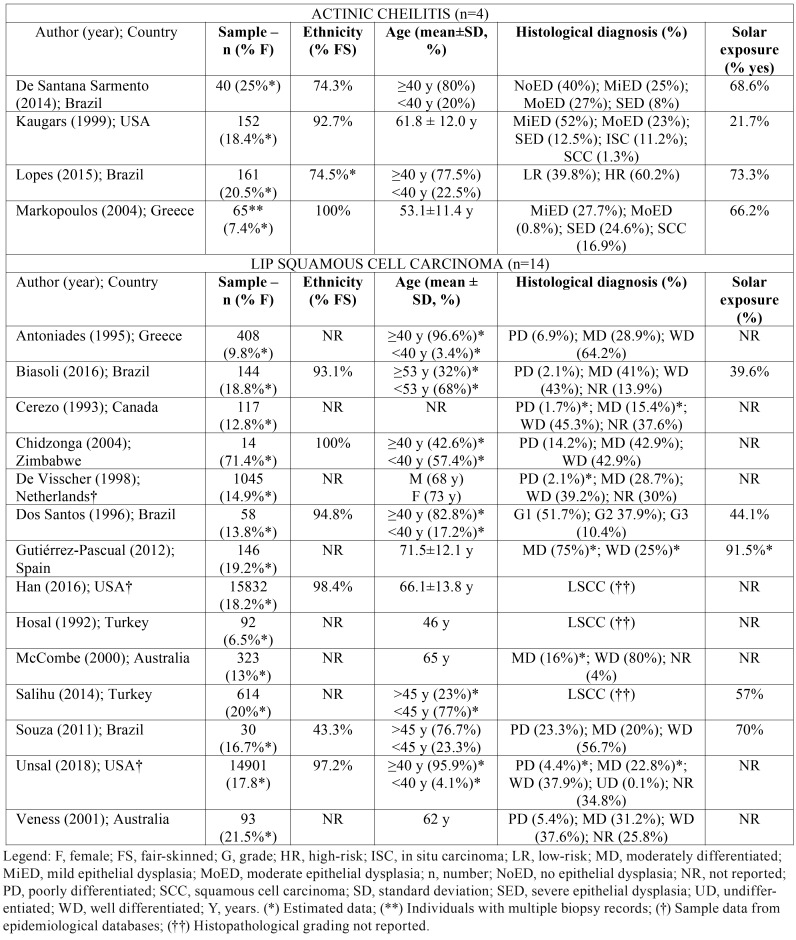
Summary of descriptive characteristics of included articles (n=18).

Overall, 418 participants (18.2% women) were enrolled in 4 studies investigating AC (13-16). Studies were published between the years of 1999-2014 and were conducted in Brazil (13,15), Greece (16), and United States of America (14). The majority of individuals were fair-skinned and older than 40 years old. In all four studies, more than 90.0% of lesions were located in the lower lip and solar exposure was positively reported by 218 (52.2%) participants. Furthermore, three studies used the WHO criteria for histological grading and SED was observed in 8% (13) to 24.6% (16) of the total amount of cases. Only one study (15) adopted the binary system of classification of epithelial dysplasia for histological grading, reporting that the majority of cases was classified as high-risk for malignant transformation (60.2%). Moreover, it is worth mentioning that histological diagnosis of LSCC was observed in 1.3% (14) and 16.9% (16) of cases clinically diagnosed as AC.

In regard to LSCC, 12 studies published between 1992-2016 investigated samples from Australia (17,18), Brazil (19-21), Canada (22), Greece (23), Netherlands (24), Spain (25), Turkey (26,27), and Zimbabwe (28), with the enrollment of 3094 (15.2% women) participants. Only 4 papers reported data concerning ethnicity (19-21,28), from which 2 reported that fair-skinned individuals composed the larger part of the sample (more than 90.0%) (19,20). Similarly, most studies reported that participants older than 40 years old were the majority of the sample, with the exception of Salihu (27), which reported 76.7% of younger individuals, and Chidzonga (28), reporting 57.4%. Lower lip was the most affected site in all studies, ranging from 83.3% (21) to 100% (17,27) of cases. Furthermore, histological grading was reported following the WHO criteria in the majority of studies; well-differentiated lip LSCC ranged from 25.0% (25) to 80.0% (17) of all cases. Only a single paper (20) followed the histologic grade proposed by Broders (29) and only 10.4% of cases were considered as Grade 3.

Regarding populations from the USA in particular, two articles investigating LSSC used national databases and evaluated data from 30733 (18.0% women) Americans (30,31). In both studies, data were collected predominantly from fair-skinned individuals (over than 97.0%) in the fourth to sixth decade of life. Lower lip was mostly affected, ranging from 77.8% (30) to 83.8% (31) of all cases. Histological grading according to the WHO criteria was reported by only one study (31) and well differentiated LSCC accounted for 37.9% of all cases.

## Discussion

The reported prevalence of LSCC is variable in the literature, ranging from 1.27 to 5.3 per 100.000 individuals (4,32), and the prevalence of AC can vary between 4.4% and 5.1% of the oral lesions (33). Most of the cases included in our sample were clinically diagnosed as AC and approximately 10% as LSCC. However, some of the cases diagnosed as AC were, in fact, LSCC in the histologic analysis, which highlight the importance of diagnosing these lesions at an early stage, since an earlier beginning of the treatment results in fewer consequences for the patient and higher survival rates (34).

The most affected site by LSCC and AC in our sample and in the studies included in the LR was the lower lip, which is frequently related in the literature as the preferential site for LSCC (35). In our sample, only four cases were located in the upper lip, from which one was LSCC (3.2%) and three were AC (3.2%). In the included studies the results were similar for AC cases; however, approximately 8% of LSCC cases were reported in the upper lip, which is considerably higher than data from the MS. Since lip vermillion semi-mucosa can be highly affected by UV exposure, it is important to emphasize prevention campaigns as relevant ways of aware a high number of individuals, especially in locations with an economy based in agriculture or livestock, such as some areas in Santa Catarina state in Brazil. This population needs to be aware of the ways to prevent AC and LSCC, such as utilizing wide-brimmed hat and lip balms with sun protection factor during labor activities and solar exposure.

None of the included studies from the LR reported data from both lesions (AC and LSCC), therefore, demographic profiles’ differences between the MS results and the LR, considering both lesions simultaneously, was not possible to be performed. Data from our survey showed that the most prevalent profile of the patients, for both AC and LSCC, was fair-skinned males, older than 40 years of age, and exposed to solar UV radiation due to labor or leisure activities. Overall, these results were similar to findings of LR; however, in regards to LSSC, few studies presented data of the ethnicity of the individuals (19-21,28,30,31) which hampered the comparison of this specific aspect of the demographic profile of individuals. Furthermore, most identified studies including LSCC did not bring data in regards to etiological factor.

Only five of the fourteen papers investigating LSCC reported data in regards to solar exposure of the individuals (19-21,25,27), still, most of these articles reported similar results to the MS.

Fair-skinned people are naturally more susceptive to UV related disorders, such as skin cancer (4,6). This fact associated with intense incidence of solar radiation, which occurs in tropical countries such as Brazil, leads to an increase in the incidence of lip cancer. Moreover, the pathology laboratory from the MS is located on a tropical island, which has a high concentration of street workers, especially in the summer season, when UV incidence rises, a fact that may increase their susceptibility to LSCC and AC. Interestingly, five (27.8%) of the included articles were also performed in Brazil and two (11.1%) in Australia demonstrating the importance of studying AC and LSCC in tropical locations, which present higher incidences of UV radiation and, therefore, higher incidences of sun exposure related diseases. In this way, health care professionals, especially in these geographic locations, need to be vigilant, since diagnosing these lesions at the earliest stage possible can provide a better prognosis and, consequently, fewer treatment-related consequences for these individuals.

It should be mentioned that the consumption of smoking tobacco was significantly higher in individuals with LSCC than in individuals with AC in the MS sample. The rates of smoking tobacco in the MS LSCC sample were similar to other studies included in the LR (21,25,27). Several cigar associated substances are linked to the development of intra-oral cancer; however, the role of these substances in the LSCC physiopathology is not clear. Some previous studies suggested that smoking may be considered an important secondary factor in LSCC, affecting both upper and lower lips equally, since there are reports of LSCC developing at the site where the cigar is placed (6). However, most of the lesions in our sample were located in the lower lip, which is commonly associated with UV damage, and rarely with the carcinogenic effect of secondary factors, such as alcohol and smoking habits. Still, these behavior habits may be in fact confounding factors in our sample, external effects (such as sun exposure) seem to have a major role in the development of LSCC rather than tobacco and alcohol consumption (36).

In regards to histopathological diagnosis, 10% of cases of LSCC in the MS were poorly differentiated, which is higher than most of the included studies from the LR. Overall, most studies reported less than 6% of LSCC as poorly differentiated and only two studies reported higher rates of poorly differentiated LSCC than MS (21,28). In addition, most cases of AC in the MS presented some degree of epithelial dysplasia, which collaborates with results from the LR. However, only four included articles in the LR presented data of AC with histopathological analysis (13-16). This fact emphasizes the need for further studies on AC clinical, demographic, and histopathological profile since there are limited data in the literature on this topic.

This study may present limitations concerning its methodology; because of the retrospective design, it was not possible to follow the patients’ clinical course. Therefore, it was not feasible to measure survival rates and evolution of AC to LSCC. LSCC survival rates vary in the literature, patients affected by this disorder seem to present better survival rates when compared to intraoral carcinomas. LSCC survival rates range between 62 and 81% (8,37,38) within 5 years after detection, additionally, the clinical stage of LSCC seems to be the main factor associated with 5-year disease-free survival (39). Despite the prolonged clinical course of these conditions, severe consequences related to the treatment can affect function, phonation, chewing, and swallowing movements. The mutilation caused by the surgical treatment affects an extra-oral region; therefore, it could lead to severe aesthetical consequences, interfering with self-image, thus profoundly affecting patients’ quality of life (40).

In conclusion, most cases of AC and LSCC in the MS were in fair-skinned individuals with solar exposure, and these data were similar to findings from the LR. It should be emphasized that most of the lesions clinically diagnosed as AC had some kind of epithelial dysplasia or, in some cases, the presence of LSCC after histopathological evaluation. Patients with a history of chronic and cumulative UV exposure should be carefully evaluated and, when lip alterations are present, biopsy and histopathological evaluation should be considered. Additionally, community-based prevention campaigns might have a marked importance in tropical countries populations, especially in the ones with a high concentration of outdoor labor occupations.
